# Perforation of the Pregnant Uterus during Laparoscopy for Suspected Internal Herniation after Gastric Bypass

**DOI:** 10.1155/2014/720181

**Published:** 2014-11-18

**Authors:** T. Mala, N. K. Harsem, S. Røstad, L. C. Mathisen, A. F. Jacobsen

**Affiliations:** ^1^Department of Gastroenterologic Surgery, Oslo University Hospital, Pb 4950 Nydalen, 0424 Oslo, Norway; ^2^Department of Gynecology and Obstetrics, Oslo University Hospital, 4950 Oslo, Norway; ^3^Department of Anaesthesiology, Oslo University Hospital, 4950 Oslo, Norway

## Abstract

We report perforations of a pregnant uterus during laparoscopy for suspected internal herniation after gastric bypass at 24 weeks of gestation. Abdominal access and gas insufflation were achieved by the use of a 12 mm optic trocar. An additional 5 mm trocar was positioned. The perforations were handled by suturing following laparotomy and mobilisation of the high located uterus. The uterine fundus was located in the subcostal area. Internal herniation was not verified. A cesarean section was made 6 weeks later due to acute low abdominal pain. During delivery the uterus was found normal. At 5 months of age the child has developed normal and seems healthy. Optical trocars should be used with caution for abdominal access during laparoscopy in pregnancy. Open access should probably be preferred in most cases. Accidental perforations of the uterine cavity may be handled in selected cases with simple closure even following the use of large trocars under close postoperative surveillance throughout the pregnancy.

## 1. Introduction

Roux-en-Y gastric bypass (RYGB) is widely applied in the treatment of morbid obesity, often in young females. Internal herniation is a potentially serious complication following RYGB and a low threshold for surgery is advised if internal herniation is suspected to relieve intestinal obstruction and the potentially compromised vascularisation [1, 2]. We report accidental perforations of the pregnant uterine cavity during laparoscopy for suspected internal herniation handled by suture of the perforations.

## 2. Case Presentation

A 35-year-old woman at 24 weeks and 4 days of gestation was admitted following 7 days with epigastric pain, postprandial vomiting, and abdominal bloating causing nutritional problems. Her history included appendectomy, RYGB 4.5 years earlier, two pregnancies before the RYGB, and laparoscopy for suspected internal herniation. Abdominal and obstetrical ultrasonography, gynaecological examination, and upper endoscopy were normal. Subacute internal herniation was suspected.

During surgery the patient was hypotensive and positioned flat and rotated to her left. A 12 mm Visiport was applied to access the abdominal cavity, 7-8 cm above the suspected fundus uteri palpated at the umbilical level. This corresponded with the anticipated level of the uterine fundus level based on age of gestation. The trocar was directed towards the subhepatic region. Intraabdominal CO_2_ gas pressure was set to 12 mmHg. Following trocar insertion, intra-abdominal fluid was observed and suspected to be induced by herniated intestines. The camera tip could be palpated in the right subcostal area below the costal margin. A 5 mm trocar was introduced at the right side superior to the first trocar. A fetal foot was then observed. Gas was exufflated and the trocars were closed. The obstetricians attended and a laparotomy extending high up in the epigastrium was made to mobilize the uterus with the trocars in the intrauterine position. The trocar entry points were identified below the tubes, more than 5 cm from the uterine fundus. The trocars were removed following placement of purshing sutures (3–0 absorbable, Vicryl). A cesarean section was not considered a viable option. A few extra sutures were made. The enteroenteroanastomosis was identified posterior to the high located and downward mobilised uterus. No pathology was identified.

Postoperatively, atosiban was given for 48 hours. Betamethasone, anti-D immunoglobulin, prophylactic cefuroxime, metronidazole, and an epidural catheter were administered. The patient remained at the intensive care unit for 2 days and subsequently 15 days at the unit for high risk pregnancies. She felt the abdomen less distended and could eat. Repeated obstetrical ultrasonographic examinations were normal during the hospital stay.

At gestational week 30, the patient experienced acute lower abdominal pain. Obstetrical ultrasonography pictured fluid in the upper uterine wall. A dissected rupture could not be excluded and a cesarean section was performed. The uterus was explored without any defects. The 1800 gram infant was in well shape. The 1 minute APGAR score was 7 and the 5 minute score was 6. However, the infant deteriorated with respiratory problems and pulmonary hypertension and was transferred to the University Hospital Neonatal Intensive Care Unit. Seven weeks later the infant was discharged home. Five months old the child has developed normally and seems healthy.

## 3. Comment

Although optical trocars allow visualization of the abdominal wall layers and thus may help avoiding inadvertent intraabdominal injuries due to a lack of vision, they should be used with caution in pregnancy [3]. At our institution Visiport is used as standard procedure for abdominal access and gas insufflation during bariatric surgery. However, the pregnant uterus does not move away easily from the tip during insertion and perforation may occur before the pressure can be relieved due to optical findings during insertion.

Open access for laparoscopy and intraoperative ultrasonography to ensure the location of the uterus fundus should be considered in pregnancy, perhaps especially in obese women. Veress needle should be used with caution in the second and third trimesters [4]. The use of Veress needle would probably also have lacerated the uterus in our patient due to the very high location of the uterus in the subcostal region. In a French consensus, open laparoscopic access above the umbilicus is recommended after 24 weeks of gestation [5].

Intrauterine CO2 insufflation may cause fetal acidosis and air embolism [6]. Potential consequences of uterine perforation include subsequent uterine rupture, amniotic fluid leakage, preterm rupture of fetal membranes, infections, preterm delivery, and laceration of the foetus or the placenta. The size of the perforation is likely of importance. Another report describes perforations of the uterus at 19 weeks of gestation by a Veress needle and a 5 mm trocar successfully closed by suturing. Premature rupture of the membranes occurred at 32 weeks of gestation [7].

Pregnant patients presenting with abdominal pain after the widely applied RYGB procedure will probably be more commonly encountered. Several reports address internal herniation in pregnancy [1, 2, 8–12]. In a review, the maternal death rate was 9% and the fetal death rate was 14%. Symptoms included abdominal pain (100%), nausea (55%), vomiting (46%), and uterine contractions (5%). Abdominal pain was severe in 2 patients (9%). Median duration of all symptoms before consulting a physician was 48 hours [12]. We speculate that the high location of the uterus may have caused the symptoms our patient experienced before surgery that was relieved after mobilisation of the uterus.

Close followup of the pregnancy after gastric bypass is recommended [13]. Focus on gastrointestinal symptoms may improve the patient awareness of the risk and signs of internal herniation. Timing of pregnancy is typically suggested to be postponed to 12–24 months after bariatric surgery due to rapid weight loss and the risk of nutritional deficiencies [13]. Whether postponing the pregnancy may affect the risk of internal herniation is not known.

In summary, other means than optical trocars should be considered for abdominal access during laparoscopy in pregnancy. Accidental perforations of the uterine cavity may be handled in selected cases with simple closure even following the use of large trocars under close postoperative surveillance throughout the pregnancy.
